# Identification of Differential Intestinal Mucosa Transcriptomic Biomarkers for Ulcerative Colitis by Bioinformatics Analysis

**DOI:** 10.1155/2020/8876565

**Published:** 2020-10-21

**Authors:** Fang Cheng, Qiang Li, Jinglin Wang, Fang Zeng, Kaiping Wang, Yu Zhang

**Affiliations:** ^1^Pharmacy, Union Hospital, Tongji Medical College, Huazhong University of Science and Technology, No. 1277 Jiefang Road, 430022 Wuhan, China; ^2^Hubei Key Laboratory of Nature Medicinal Chemistry and Resource Evaluation, Tongji Medical College, Huazhong University of Science and Technology, 430030 Wuhan, China

## Abstract

**Background:**

Ulcerative colitis (UC) is a complicated disease caused by the interaction between genetic and environmental factors that affect mucosal homeostasis and triggers inappropriate immune response. The purpose of the study was to identify significant biomarkers with potential therapeutic targets and the underlying mechanisms.

**Methods:**

The gene expression profiles of GSE48958, GSE73661, and GSE59071 are from the GEO database. Differentially expressed genes (DEGs) were screened by the GEO2R tool. Next, the Database for Annotation, Visualization and Integrated Discovery (DAVID) was applied to analyze gene ontology (GO) and Kyoto Encyclopedia of Genes and Genomes (KEGG) pathway. Then, protein-protein interaction (PPI) was visualized by Cytoscape with Search Tool for the Retrieval of Interacting Genes (STRING).

**Results:**

There were a total of 128 common DEGs genes, including 86 upregulated genes enriched in extracellular space, regulation of inflammatory response, chemokine-mediated signaling pathway, response to lipopolysaccharide, and cell proliferation, while 42 downregulated genes enriched in the integral component of the membrane, the integral component of the plasma membrane, apical plasma membrane, symporter activity, and chloride channel activity. The KEGG pathway analysis results demonstrated that DEGs were particularly enriched in cytokine-cytokine receptor interaction, TNF signaling pathway, chemokine signaling pathway, pertussis, and rheumatoid arthritis. 18 central modules of the PPI networks were selected with Cytotype MCODE. Furthermore, 18 genes were found to significantly enrich in the extracellular space, inflammatory response, chemokine-mediated signaling pathway, TNF signaling pathway, regulation of cell proliferation, and immune response via reanalysis of DAVID.

**Conclusion:**

The study identified DEGs, key target genes, functional pathways, and pathway analysis of UC, which may provide potential molecular targets and diagnostic biomarkers for UC.

## 1. Introduction

Ulcerative colitis (UC) is one of the major clinical phenotypes of inflammatory bowel disease (IBD), a complicated disease caused by the interaction between genetic and environmental factors that affect mucosal homeostasis and trigger inappropriate immunity response [1]. The incidence rate of UC in western countries has been increasing steadily and exhibiting a further increasing incidence in recent years in China [2]. In addition, UC often develops into a long-term health condition, with multiple complications, and reduces the patients' health-related quality of life [3].

Although great progress has been made in understanding the pathophysiology of UC, early diagnosis, therapeutic intervention, and the potential pathogenesis remain to be fully elucidated. At present, UC is mainly diagnosed through colonoscopy and biopsy, which brings great pain to UC patients. Furthermore, the diagnosis may be delayed for several years, and it is difficult to make even for trained physicians [4]. Therefore, it is very important to formulate a more accurate diagnosis and effective treatment strategies to improve the prognosis of patients.

Therefore, early and accurate diagnosis of biomarkers could help clinicians to improve the treatment of individual patients. Furthermore, the biomarkers can contribute to predict the disease courses and thus identify patients who require intensive treatment. Patients with a low risk of morbidity may avoid the medication usage accompanied by the risk of adverse events. Moreover, disease recognition and specific biomarkers could be applied to identify the biological pathways involved in disease development and treatment. Deepening the overall understanding of the disease mechanism can promote the development of prevention and treatment strategies in the future. Therefore, the clinical application of a set of biomarkers represents a potentially valuable tool for diagnosis and prognosis.

Currently, there are no effective biomarkers or commercial tests for early diagnosis of UC in clinical practice. The bioinformatics analysis has been widely used for exploring the molecular mechanisms of various diseases [5–7], which contribute to identify novel biomarkers able to improve both diagnostic and prognostic strategies of UC.

In recent years, a large number of candidate genes, RNA sequencing, and mucosal microarray studies of UC patients have been published [8, 9]. Several key genes and candidate biomarkers of UC, such as Cadherin 11, Hepatocyte nuclear factor 4 alpha (HNF4*α*), Intercellular adhesion molecule 1 (ICAM1), and Ring finger protein 186 (RNF186) have been identified by bioinformatics analyses [10–12]. There were a variety of cellular pathways considered to be related to UC, including epithelial repair, barrier function, immune regulation, autophagy, microbial defense, cell proliferation, and apoptosis [13, 14]. However, because these markers are also in the presence of various inflammatory conditions, the sensitivity and specificity have not been sufficient for successfully implementing in clinical. Therefore, there is an urgency to identify novel biomarkers for the early diagnosis of UC.

In this study, we downloaded GSE48958, GSE73661, and GSE59071 from the Gene Expression Omnibus (GEO) database. Second, we applied the GEO2R online tool and the Venn diagram software to obtain the commonly differentially expressed genes (DEGs) in the three datasets. Third, Gene Ontology (GO) and Kyoto Encyclopedia of Genes and Genomes (KEGG) analyses conducted in the DAVID database were used to determine the functional enrichment and important pathways related to the DEGs. Then, we established a protein-protein interaction (PPI) network and then applied Cytotype MCODE (Molecular Complex Detection) to identify the core genes and significant modules. In conclusion, the present study provided some additional useful biomarkers, which may facilitate an accurate diagnosis and provide potential therapeutic targets for UC patients.

## 2. Materials and Methods

### 2.1. Acquisition of Data of Gene Expression Profiles

NCBI-GEO (https://www.ncbi.nlm.nih.gov/geo/) is regarded as a free public database of microarray/gene profile, and we obtained the gene expression profile of GSE48958, GSE73661, and GSE59071 in UC and normal tissues. The microarray data of GSE48958, GSE73661, and GSE59071 were all on account of GPL6244 Platforms ([HuGene-1_0st] Affymetrix Human Gene 1.0 ST Array). The present study focused on colonic mucosal biopsies, which included 7 colonic tissues and 8 normal controls, 23 colonic biopsies and 12 normal tissues, and 97 UC patients and 11 controls, respectively.

### 2.2. Screening of DEGs

GEO2R was used for data preprocessing and applied to screen DEGs between the following groups: UC vs. control group. The ∣logFC | >2 and adjusted *P* value <0.05 were selected as the threshold for each group. The gene differential analysis from the three microarrays was conducted with volcano plots. Then, the extracted raw data were checked in the Venn software online to detect the common DEGs among the three datasets. The DEGs with log FC > 0 were considered as upregulated genes, while log FC < 0 was considered as downregulated ones.

### 2.3. GO Enrichment and KEGG Pathway Analyses of DEGs

In order to analyze the screened DEGs at the functional level, GO function enrichment was performed using the DAVID online tool (https://david.ncifcrf.gov/summary.jsp). And the KEGG pathway analysis was downloaded from the KEGG database (https://www.kegg.jp/). *P* < 0.05 was set as the cutoff criterion.

### 2.4. PPI Network Analysis

To further visualize and evaluate interactions among DEGs, the STRING online database (https://string-db.org/cgi/input.pl) in Cytoscape was applied to examine the potential correlation between these DEGs with the node association confidence score > 0.4.

### 2.5. Module Analysis

The plug-in Molecular Complex Detection (MCODE) was used to identify the hub gene in functional networks in Cytoscape. The Biological Networks Gene Ontology tool (BINGO) plug-in in Cytoscape was conducted to the GO network of hub genes from the PPI network. *P* < 0.05 was considered a significant difference.

## 3. Results

### 3.1. Identification of DEGs

**Figure 1:**
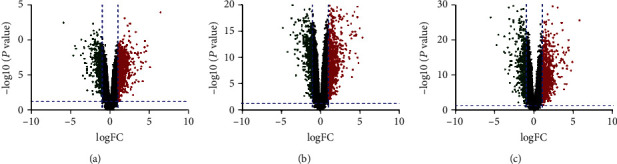
Volcanic plots of GSE59071, GSE48958, and GSE73661 differential genes: (a) GSE59071; (b) GSE48958; (c) GSE73661. Data points in red represent upregulated and in green represent downregulated genes. Genes without any significant difference are in black.

**Figure 2:**
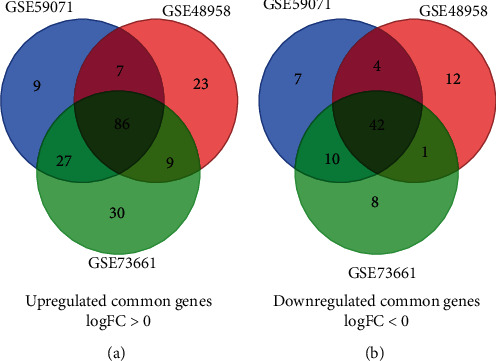
Authentication of 128 common DEGs in the three datasets through the Venn diagram software: (a) upregulated genes; (b) downregulated genes.

First, we analyzed all the genes from GSE59071, GSE48958, and GSE73661 in Figure 1. Then, we, respectively, extracted 192, 184, and 213 DEGs from GSE59071, GSE48958, and GSE73661 with the threshold of *P* < 0.05 and ∣logFC | >2. Furthermore, the selected common DEGs are shown in Figure 2. A total of 128 common DEGs were detected, including 86 upregulated genes (logFC > 0) and 42 downregulated genes (logFC < 0) (Table 1).

### 3.2. GO Enrichment and KEGG Pathway Analyses of DEGs

**Figure 3:**
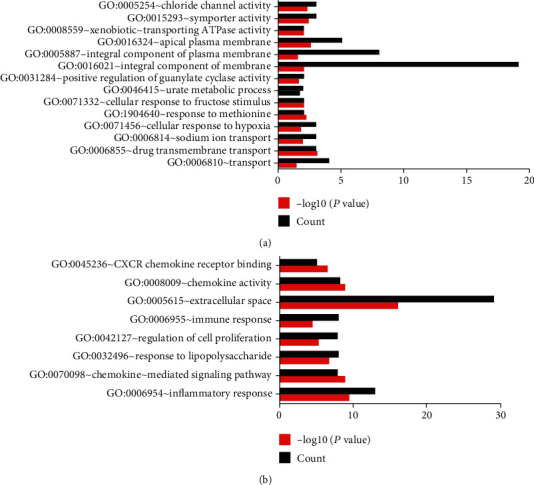
GO analysis of the common DEGs: (a) upregulated genes; (b) downregulated genes.

**Figure 4:**
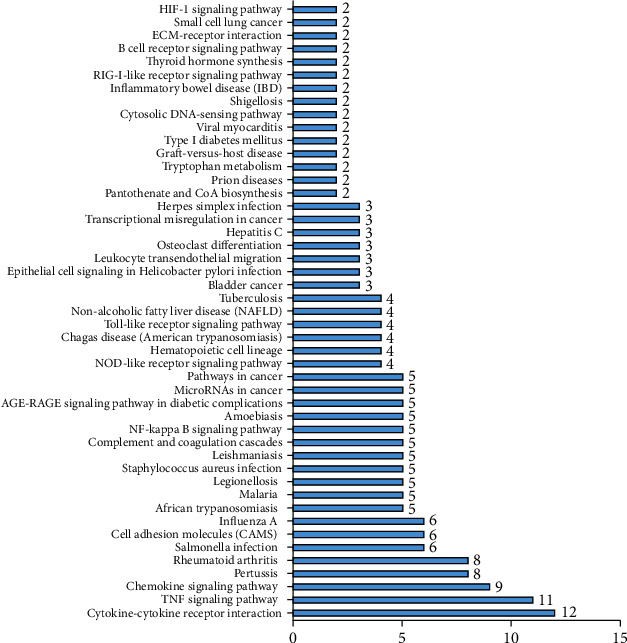
KEGG pathway functional enrichment analysis of the common DEGs.

GO analysis includes molecular function, biological processes, and cell composition. The GO enrichment analysis of upregulated DEGs is presented in Figure 3. The results indicated that upregulated DEGs were particularly enriched in extracellular space, regulation of inflammatory response, chemokine-mediated signaling pathway, response to lipopolysaccharide, cell proliferation, immune response, and chemokine activity (Figure 3(a)). However, the downregulated DEGs are the integral components of the membrane, the integral component of the plasma membrane, apical plasma membrane, transport, drug transmembrane transport, apical plasma membrane, symporter activity, chloride channel activity (Figure 3(b)). The KEGG pathway analysis results are shown in Figure 4, which demonstrated that DEGs were particularly enriched in cytokine-cytokine receptor interaction, TNF signaling pathway, chemokine signaling pathway, pertussis, and rheumatoid arthritis (*P* < 0.05).

### 3.3. PPI Network Integration

**Figure 5:**
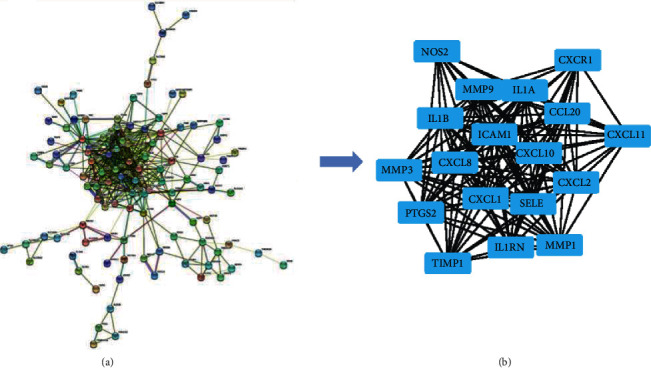
Common DEGs PPI network constructed by the STRING online database and module analysis. (a) PPI network complex. The nodes meant proteins. (b) Module analysis via the Cytoscape software.

The STRING database was applied to investigate the PPI networks of these DEGs. The result is shown in Figure 5(a), in which the PPI network of the overlapping DEGs consisted of 121 nodes and 450 edges.

### 3.4. Module Analysis of the PPI Network

**Figure 6:**
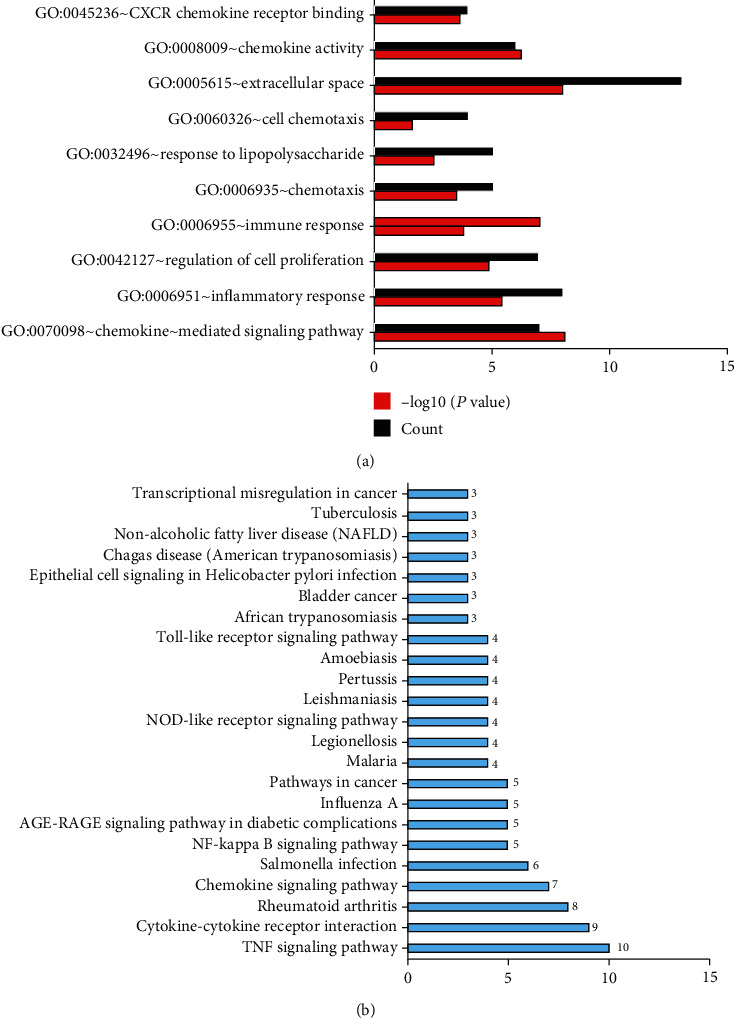
GO and KEGG pathway functional enrichment analysis of the 18 hub genes: (a) GO analysis; (b) KEGG pathway functional enrichment analysis.

18 central node modules of the PPI networks were selected with Cytotype MCODE (Figure 5(b)). And the GO enrichment and KEGG pathway analysis of DEGs in the respective modules were analyzed. The result of the GO enrichment analysis is shown in Table 2 and Figure 6(a), which demonstrated that genes in the module were mainly related to extracellular space, inflammatory response, chemokine-mediated signaling pathway, regulation of cell proliferation, and immune response. The KEGG pathway analysis revealed that these genes were mainly associated with the TNF signaling pathway, cytokine-cytokine receptor interaction, rheumatoid arthritis, and chemokine signaling pathway (Figure 6(b)).

## 4. Discussion

The etiology of UC has involved a complex interaction between environmental factors, infectious agents, and genetic susceptibility, which results in the impairment of mucosal immune response and barrier function against the intestinal microbiota [15]. For example, among the genetic factors associated with the UC etiology, variants in the autophagy-related genes have been identified. Recent studies have shown that autophagy played a key role in maintaining intestinal homeostasis and regulating the interaction between gut microbiota and innate and adaptive immunity [16, 17]. Therefore, the research progress of UC molecular mechanism based on microarray technology can provide potential targets for the diagnosis and treatment of UC.

In the present study, 128 DEGs were screened in the UC samples, including 86 upregulated and 42 downregulated genes. The results of the GO analysis indicated that DEGs were particularly enriched in extracellular space, regulation of inflammatory response, chemokine-mediated signaling pathway, response to lipopolysaccharide, cell proliferation, immune response, and integral component of membrane. Previous studies have indicated that the abovementioned GO terms are potentially significant events in the pathogenesis of UC. For example, the regulation of inflammatory response, chemokine-mediated signaling pathway, response to lipopolysaccharide, cell proliferation, and immune response have roles in the pathogenesis of UC [18, 19]. Furthermore, there was increasing evidence showing that extracellular space plays a pathogenic role in UC [20]. Excessive unneutralized hydrogen peroxide generated in the colonic epithelial cells due to aberrant cell metabolism diffuses through cell membranes to the extracellular space where it is converted to the highly destructive hydroxyl radical, which results in oxidative damage to the structure of the colonic epithelial barrier.

Furthermore, the KEGG pathway analysis revealed that the common DEGs were particularly enriched in cytokine-cytokine receptor interaction, TNF signaling pathway, chemokine signaling pathway, pertussis, and rheumatoid arthritis. Multiple immune and inflammatory signaling pathways, including the cytokine-cytokine receptor interaction, TNF signaling pathway, and chemokine signaling pathway, are activated and involved in the process of intestinal inflammation [8, 21]. Previous studies have demonstrated that the immune-inflammatory response pathway was closely associated with the pathogenesis of UC [22, 23], which is mediated by a complex and dynamic relationship between immune cells and cytokines. For instance, the pathways include cytokine-cytokine receptor interaction, and the TNF signaling pathway was significantly associated with the occurrence and development of UC [24]. Moreover, a variety of parenteral diseases are related to IBD, which are common complications of IBDs and are associated with the impairment quality of life [25, 26]. An increased prevalence of rheumatoid arthritis has been reported in UC patients. Previous studies have also demonstrated that the pertussis vaccine was immunogenic and safe in pediatric patients with UC, particularly when used in combination with anti-TNF-*α* agents [27, 28].

Subsequently, UC with highly relevant pathway and enriched genes were selected to construct the PPI network; then, the potential key genes were identified. The 18 central hub genes included nitric oxide synthase 2 (NOS2), Matrix metalloproteinase 1 (MMP1), Matrix metalloproteinase 3 (MMP3), Matrix metalloproteinase 9 (MMP9), Tissue inhibitors of matrix metalloproteinase 1(TIMP1), chemokine (C-C motif) ligand 20 (CCL20), intercellular cell adhesion molecule-1(ICAM1), motif chemokine receptor 1 (CXCR1), Chemokine (C-X-C motif) ligand 1 (CXCL1), Chemokine (C-X-C motif) ligand 2 (CXCL2), Chemokine (C-X-C motif) ligand 8 (CXCL8), Chemokine (C-X-C motif) ligand 10 (CXCL10), Chemokine (C-X-C motif) ligand 11 (CXCL11), E-selectin (SELE), Interleukin-1 alpha (IL-1*α*), interleukin-1 beta (IL-1*β*), interleukin IL-1 receptor antagonist (IL1RN), and prostaglandin-endoperoxide synthase 2 (PTGS2), which were mainly associated with the inflammatory response.

NOS2 induction accounts for large nitric oxide amounts that promote oxidative stress. The LPS-induced inflammatory responses are often accompanied by a high level of nitric oxide (NO) through a macrophage expression of the inducible form of NOS2 in microglial cells [29, 30].

CXCLs, a class of small cytokines or signal proteins, play important roles in inducing directed chemotaxis of nearby reactive cells. Some chemokines are considered to be proinflammatory cytokines, which can induce cells of the immune system to enter the infection site during the immune response [31]. With regard to CXCL8, it is an effective inflammatory chemoattractant and neutrophil activator. CXCL10, also known as interferon-inducible protein-10 (IP-10), has been proved to play a significant role in leukocyte homing to inflamed tissues [32].

CXCR1/2 belongs to the G-protein coupled receptor family, which is expressed on monocytes, neutrophil, and other leukocytes. Some scholars pointed out that CXCR1 was involved in the pathogenesis of IBD [33]. CXCL8 exerts its effects on neutrophils by binding with CXCR1/2, which have been proved to play a vital role in promoting neutrophil activation and recruitment to the site of inflammation [34]. Therefore, successfully preventing the interaction between CXCL8 and CXCR1/2 could effectively limit the recruitment of neutrophils and slow down the inflammation response.

ICAM-1 is a member of the immunoglobulin superfamily of adhesion molecules. When stimulated by inflammatory cytokines (such as IL-1 and TNF-*α*) and endotoxin, it can be expressed on many cells. The adhesion molecules ICAM-1, associated with macrophage infiltration, are directly related to cell migration in inflamed colonic tissue [35]. The upregulation of ICAM-1 expression promotes the infiltration of inflammatory cells to the inflammatory site and releases more inflammatory mediators and cytokines [36]. The interaction of ICAM-1 and inflammatory cytokines aggravates the formation of malignant circulation of inflammation.

The IL1RN gene is a protein member encoding the IL-1 cytokine family, which can inhibit IL-1*α* and IL-1*β* activities and regulate a variety of related immune-inflammatory responses [37]. Some studies have reported a significant correlation with CD and UC susceptibility and treatment outcomes [38]. Interestingly, the IL-1RN∗2 was associated with decreased levels of IL-1RA protein and IL-1RN mRNA in the colonic mucosa of UC patients [39].

SELE, also known as E-selectin, is one of the members of the selectin family. It mainly exists in endothelial cells and has a wide distribution of ligands. After SELE is combined with the ligand, it can promote the leukocytes to enter the inflammation area through the blood vessel wall and promote the inflammatory response and aggravate the infiltration of inflammatory cells in the local airway [40]. It has been reported that the level of SELE in patients with asthma is significantly increased, and the content of SELE is positively correlated with the content of IgE [41].

MMPs is a family of zinc-dependent endopeptidases, which plays a key role in tumor invasion and metastasis. MMPs are transcriptionally upregulated by proinflammatory cytokines, and both the mRNA and protein levels of some MMPs have been confirmed to be upregulated in inflamed mucosa or serum of IBD patients [42]. TIMPs is a group of small secreted glycoprotein that could inhibit the activation of MMPs results in the accumulation of ECM products [43]. Since TGF-*β* signaling is regulated by the balance between TIMPs and MMPs, the sustained activation of TIMPs may have a feedback inhibition effect on the transcription of TGF-*β*. It has been shown that amphiregulin promotes the invasion of different malignant cells through altering the MMPs/TIMPs balance [44].

CCL20 is a chemokine mainly expressed in peripheral immune organs or tissues. It interacts with its specific receptor chemokine receptor 7 to mediate the inflammatory response and promote the expression of MMP9 [45, 46]. A significant association of MMP9, TIMP1, CXCL10, and CCL20 with UC correlated CRC development and thus may be indicative for evaluating the prognosis of CRC [47, 48]. MMP9 can degrade the basement membrane and the matrix surrounding the tumor, help it break through the matrix barrier, and contribute to tumor invasion and metastasis. At the same time, MMP9 plays a key role in the formation, invasion, and metastasis of CRC by promoting neovascularization, capillary proliferation, and tumor cell growth and proliferation. TIMP1 can not only inhibit the hydrolysis of matrix protein but also promote the growth and metastasis of tumor cells [49]. Once the dynamic balance between TIMP1 and MMP9 is broken, it may promote tumor invasion and metastasis. Previous studies have shown that MMP1 and TIMP1 are involved in the development of UC, which provides a basis for the treatment of UC [50]. CXCL10 may recruit the leukocytes to inflammation sites. However, the latest report has indicated that CXCL10 may promote the development of colon cancer by promoting cytokine-mediated mucosal damage and inflammation [51].

Over the past decades, with the development of pathology, multiomics, and bioinformatics, the role of cytokines and cell adhesion molecules has been confirmed in the pathogenesis of UC. Until recently, the anticytokine therapy, such as tumor necrosis factor (TNF) antagonists (adalimumab, golimumab, and infliximab) and anti-a4b7 antibody (vedolizumab), has been approved in the management of UC patients [52]. However, the results of clinical practice showed that these biologics are only effective in a subgroup of patients with UC [53]. Indeed, a significant number of patients experience inflammation relapse after cessation of treatment [54]. Therefore, the pathogenesis of UC is complicated and the therapeutic effect may vary between patients. It is important to continuously reveal the potential pathogenesis and promote effective drug development.

The purpose of the research on genes and biomarkers related to UC has been the possibility to predict the treatment response and ultimately minimize and prevent possible adverse reactions. On the other hand, it is committed to promoting the development of new therapeutic drugs. A recent meta-analysis had reported a weak association of TLR2, TLR4, TLR9, TNFRSF1A, IFNG, IL6, and IL1B with the treatment response to infliximab [55].

The development of new molecules targeting simultaneously multiple cytokines has been proven to be effective in UC. The discovery of the Janus kinase (JAKs) family of tyrosine kinases elucidated their role in cytokine signaling pathways, which have been identified as potential therapeutic targets of UC [56]. Tofacitinib, a pan-JAK inhibitor, has been recently approved for the treatment of moderate-to-severe UC [57].

It was worth noting that this study has limitations. In this study, the results of microarray expression profiling were analyzed using bioinformatics methods and were not verified by reverse transcription-quantitative (RT-q) PCR. Therefore, a large number of clinical samples and future studies for experimental verification are required.

## 5. Conclusion

In summary, the study used a comprehensive analysis method to identify DEGs, as well as unique biological functions and pathways of UC, thereby enhancing the current understanding of the pathogenesis of UC. Moreover, these results may provide potential biomarkers for the early and accurate diagnosis of UC, as well as potential therapeutic targets for the development of novel UC treatments.

## Figures and Tables

**Table 1:** 86 upregulated commonly differentially expressed genes (DEGs) and 42 downregulated genes in the UC compared to normal tissues.

DEGs	Genes name
Upregulated	LCN2 CDH3I GHM///IGHG1///IGHD///IGHA2///IGHA1///IGHA2///IGHA1///IGH OSMR TNIP3 CXCL10 ITGA5 C4BPA SAA2 ALDOB TNFAIP6 MXRA5 STEAP4 S100A12 IDO1 GBP5 MMP7 LOC102723407///IGHV431///IGHM///IGHD///IGHA2///LOC102723407///SKAP2///IGHA2///IGHA1///IGH NOS2 SERPINB5 APOL1 SLC7A11 SERPINB3 TNC FCGR3B///FCGR3A UBD VSIG1 VNN2 IL1A AQP9 MMP3 SERPINA3 ICAM1 CCL18 REG3A SOCS3 MMP10 DUOX2 SPINK4 CXCL13 SELP TIMP1 PHLDA1 REG1B PLEK S100A8 CXCR1 ABCA12 PI3 SLC6A14 IGK///IGKC DAPP1 TGM2 CFB C2 CXCL1 CHI3L1 KYNU TCN1 SERPINB7 CCL20 GBP4 PIM2 CD55 CXCL11 MMP1 OLFM4 IGFBP5 REG4 PTGS2 MMP12 SELL IRAK3 CXCL8 CXCL2 CLDN1 REG1A IL1B SELE DMBT1 DUOXA2 CD79A IL1RN MMP9 C3 VNN1
Downregulated	SLC38A4 CLCA1 TMIGD1 MEP1B PPARGC1A OTOP2 CLDN8 TRPM6 HNF1A-AS1 MT1M ABCG2 BMP3///BMP3 PRKG2 CYP2B6 HMGCS2 SLC26A2 APOBEC3B TMEM236 PCK1 GUCA2A PADI2 SLC16A9 SLC30A10 GUCA2B ADH1C CWH43 PBLD BEST4 CA1 CYP2B7P TMEM63C ABCB1 AQP8 CHP2 BRINP3 SLC22A5 SLC17A4 B4GALNT2 UGT2A3 ACSF2 PHLPP2 DHRS11

**Table 2:** Gene ontology analysis of 18 central hub genes in UC.

Category	Term	Count	*P* value	Genes	--log10 (*P* value)
GOTERM_BP_DIRECT	GO:0070098~chemokine-mediated signaling pathway	7	8.79E-12	CCL20, CXCL2, CXCR1, CXCL8, CXCL11, CXCL10	7.977397
GOTERM_BP_DIRECT	GO:0006954~inflammatory response	8	3.40E-09	CCL20, PTGS2, CXCL2, CXCL8, NOS2, CXCL11, CXCL10	5.389683
GOTERM_BP_DIRECT	GO:0042127~regulation of cell proliferation	7	1.41E-08	PTGS2, CXCL2, CXCL8, NOS2, CXCL11, CXCL10	4.772917
GOTERM_BP_DIRECT	GO:0006955~immune response	7	1.42E-07	CXCL2, IL1B, CXCL8, CXCL11, IL1A, CXCL10	3.770405
GOTERM_BP_DIRECT	GO:0006935~chemotaxis	5	3.20E-07	CCL20, CXCR1, CXCL11, CXCL10	3.415649
GOTERM_BP_DIRECT	GO:0032496~response to lipopolysaccharide	5	2.71E-06	CXCL2, CXCL8, CXCL11, CXCL10	2.488617
GOTERM_BP_DIRECT	GO:0060326~cell chemotaxis	4	2.42E-05	CCL20, CXCL2, CXCL10, ICAM1,	1.537263
GOTERM_CC_DIRECT	GO:0005615~extracellular space	13	1.26E-11	CCL20, MMP9, IL1RN, CXCL2, IL1B, CXCL8,CXCL11,SELE,IL1A, CXCL10, TIMP1	7.981942
GOTERM_MF_DIRECT	GO:0008009~chemokine activity	6	7.11E-10	CCL20, CXCL2, CXCL8, CXCL11, CXCL10	6.189676
GOTERM_MF_DIRECT	GO:0045236~CXCR chemokine receptor binding	4	2.98E-07	CXCL2, CXCL8, CXCL10	3.567109

## Data Availability

The data used are available in https://www.ncbi.nlm.nih.gov/geo/.
